# A cross-sectional analysis of traditional medicine use for malaria alongside free antimalarial drugs treatment amongst adults in high-risk malaria endemic provinces of Indonesia

**DOI:** 10.1371/journal.pone.0173522

**Published:** 2017-03-22

**Authors:** Dwi Linna Suswardany, David W. Sibbritt, Sudibyo Supardi, Jerico F. Pardosi, Sungwon Chang, Jon Adams

**Affiliations:** 1 Australian Research Centre in Complementary and Integrative Medicine (ARCCIM), Faculty of Health, University of Technology Sydney, Sydney, New South Wales, Australia; 2 Universitas Muhammadiyah Surakarta, Central Java, Indonesia; 3 National Institute of Health Research and Development, Ministry of Health, Indonesia; 4 School of Public Health and Community Medicine, University of New South Wales, Australia; Institute of medical research and medicinal plant studies, CAMEROON

## Abstract

**Background:**

The level of traditional medicine use, particularly Jamu use, in Indonesia is substantial. Indonesians do not always seek timely treatment for malaria and may seek self-medication via traditional medicine. This paper reports findings from the first focused analyses of traditional medicine use for malaria in Indonesia and the first such analyses worldwide to draw upon a large sample of respondents across high-risk malaria endemic areas.

**Methods:**

A sub-study of the Indonesia Basic Health Research/Riskesdas Study 2010 focused on 12,226 adults aged 15 years and above residing in high-risk malaria-endemic provinces. Logistic regression was undertaken to determine the significant associations for traditional medicine use for malaria symptoms.

**Findings:**

Approximately one in five respondents use traditional medicine for malaria symptoms and the vast majority experiencing multiple episodes of malaria use traditional medicine alongside free antimalarial drug treatments. Respondents consuming traditional medicine for general health/common illness purposes every day (odds ratio: 3.75, 95% Confidence Interval: 2.93 4.79), those without a hospital in local vicinity (odds ratio: 1.31, 95% Confidence Interval: 1.10 1.57), and those living in poorer quality housing, were more likely to use traditional medicine for malaria symptoms.

**Conclusion:**

A substantial percentage of those with malaria symptoms utilize traditional medicine for treating their malaria symptoms. In order to promote safe and effective malaria treatment, all providing malaria care in Indonesia need to enquire with their patients about possible traditional medicine use.

## Introduction

Malaria remains a significant public health challenge in Indonesia and of particular prominence in the Eastern regions of the country [1]. While the cumulative probability of malaria death in Indonesia has decreased from 29 to 3.8 cases per 1000 population from 1980 to 2010 [2], the Indonesian mortality statistics are believed to be under-reported and underestimated due to incomplete and inaccurate death statistics, as well as incomplete coverage [3–5].

Artemisinin-based Combination Therapies (ACTs) are the treatment of choice for uncomplicated *P*. *falciparum* malaria internationally [6]. Such antimalarial drugs are generally available for free in Indonesia via conventional health centers either in the form of hospitals, government-mandated community health clinics located across Indonesia or Puskesmas (Indonesian: Pusat Kesehatan Masyarakat, English: Community Health Centre) and other smaller facilities associated with Puskesmas. While substantial numbers of those diagnosed with malaria by conventional providers do receive antimalarial drugs [7,8] the availability of such treatment in conventional health centers can vary [3,9].

Delays in seeking care, obtaining a diagnosis and receiving appropriate treatment are all associated with fatal malaria [10]. Early diagnosis and prompt treatment for malaria should occur within 24 hours of the onset of symptoms to decrease the risk of severe complications and onward transmission which may occur within a few hours for falciparum malaria [6]. However, research suggests Indonesians do not always seek timely treatment for malaria or febrile illness. Most people (66%) delayed visiting a conventional health center for malaria-related illness for at least the first three days of their fever [11] and 3% waited until ten days after the onset of their malaria symptoms before seeking conventional health center treatment [12]. In some cases, malaria patients may undertake self-treatment including the use of traditional medicine (TM) [13,14].

The Indonesian Traditional Medicine refers to Jamu, a specific Javanese term which is predominantly herbal medicine made from natural materials, such as plant material including roots, bark, flowers, seeds, leaves and fruits. Animal materials are also often used, such as honey, milk, and eggs. It is commonly embraced in Indonesia to both maintain health [15] and treat specific health problems [16,17]. Latest figures estimate a national prevalence of TM use for general purposes/maintaining health in Indonesia at just under 30% of the general population in the last five years [18]. Early empirical work has identified that 4.47% of the Indonesian population use herbal medicine every day and 17.4% use self-made herbal medicine every day [19,20]. Meanwhile, a higher prevalence of TM use has been reported among Sundanese Tribe villagers in Indonesia (albeit from a much smaller, localized sample), with almost two-third of 70 households using TM for various illnesses, such as fever, typhus, hepatitis, and postpartum remedy [21]. TM use has also been shown to be popular for personal use among Indonesian medical students and physicians [22–24].

There have been a few studies reporting the reasons for using TM for malaria treatment and the type of medicinal plants used for malaria treatment. People used Jamu for malaria treatment, specifically for treating fever, chill, and other symptoms of malaria [25]. Common reasons for using traditional medicine for malaria varied from cost consideration, availability and accessibility, perceived effectiveness, low side effect, and faith in traditional medicine [13,26–28]. Meanwhile, the type of Jamu specifically used by Indonesians for treating malaria and were reported as having anti-plasmodium activity on Plasmodium falciparum were *Caesalpinia crista Linn*. (*Fabaceae*)/or commonly known as *“bagore”* in Indonesia, *meniran (Phyllanthus urinaria)*, and *Carica papaya* extract [29–32]. In Indonesia, traditional medicines were self-made or were purchased from local Jamu vendors who sell Jamu in a store or door to door on foot or by motorcycle or even car [33]. TM for malaria can also be purchased from traditional healers or *dukun* [13]. Previous studies reported preferences using TM for malaria treatment attributable to the benefits of TM for treating fever, and the decision to use TM for malaria was based on family or neighbor’s experiences [25].

There is evidence amongst Asia-Pacific countries that are initially practicing home remedies (including TM) followed by consultation with TM healers often leads to delays in seeking treatment from a conventional health center [34–36]. Unfortunately, TM use for malaria and amongst populations in malaria-endemic locations across all low-to-middle income countries in the Asia-Pacific remains significantly under-researched, and we still know very little about TM use in Indonesia including the factors that may influence the use of TM for malaria treatment [37]. In direct response to this significant research gap, this paper reports findings from the first focused analyses of TM use for malaria in Indonesia and the first such analyses worldwide to examine TM use for malaria drawing upon a large sample (n = 12,226).

## Materials and methods

### Study area and design

The analyses presented in this paper are based on data from the Indonesia Basic Health Research (Riskesdas/*Riset Kesehatan Dasar*) drawing specifically upon the 2010 Riskesdas survey, conducted by the National Institute of Health Research Development (NIHRD), Ministry of Health Indonesia. Riskesdas was designed to examine various determinants affecting community health as well as measure progress towards Millennium Development Goals (MDGs) [7]. Riskesdas 2010 was a cross-sectional survey conducted on a civilian non-institutionalized household population of Indonesia who resided in 33 provinces (n = 241,946). Data were collected by interviewing household heads and household members based on structured questionnaires. Adults present at the time of the interview were asked to respond for themselves. Proxy responses are accepted for adults who were physically or mentally incapable of responding [7].

### Sample

The research reported here constitutes a sub-study of the Riskesdas 2010. It focused on 12,226 adults aged 15 years and above who resided in high-risk malaria-endemic provinces (i.e. 28 provinces out of 33 provinces in Indonesia) as defined by the World Malaria Report 2013 [38] with an Annual Parasites Index/API equal to 1 case or more per 1000 population. Sampling was conducted using a multi-stage stratified probability sampling method.

### Variables

The dependent variable reported here is ‘the use of TM for treating malaria symptoms.' Participants were asked whether they did or did not use TM for treating malaria symptoms in the past month. TM in this study refers to medicinal plants or Jamu. The malaria symptoms were defined as recurring cycles of fever with chills or increasing and decreasing body temperature, with or without a headache, sweating, nausea, and vomiting [39].

There were 40 independent variables used in this study to profile the characteristics of Indonesian adults who used TM for treating malaria symptoms. These were included demographic characteristics, malaria status, awareness of the availability of health services nearby, residential locations, and home environment characteristics. There were three different Malaria status statuses. First, those people who were diagnosed as having malaria in the last one month or one year; second, having malaria symptoms in the last one month; and third, not having any diagnosis or symptoms of malaria.

### Statistical analysis

All analyses were conducted using STATA 13.1. The bivariate association between the independent variables and the use of TM for treating malaria symptoms were assessed by chi-square test and two-sample t-test where appropriate. Any variables with an association with TM usage for malaria symptoms (p < 0.25 in the bivariate analyses) were included in the logistic regression modeling [40]. The final model was determined using backward stepwise selection. Due to the large sample size, statistical significance was set at p < 0.005.

### Ethical approval

Approval for the sub-study reported here was obtained from the Human Research Ethical Committee of University of Technology Sydney (UTS HREC 2014000083). Approval for initial Riskesdas 2010 data collection was obtained from the National Indonesia Health Research Development (NIHRD), Ministry of Health Indonesia (No. LB.03.04/KE/928/2010). Informed written consent was obtained from all individuals participating in the questionnaire-based interviews.

## Results

The distribution of socio-demographic and lifestyle characteristics of adult Indonesians by the use of TM for malaria symptoms are presented in Table 1. The respondents who used TM for treating malaria symptoms were more likely to be male (p = 0.0031), married (p < 0.0001), older (p < 0.0001), currently smoke (p = 0.0003), have less than 9 years of education (p < 0.0001) and be a farmer (as their main occupation) (p < 0.0001) compared to those who did not use TM for malaria symptoms. Further, TM users for malaria symptoms were more likely to be current users of TM for more general health conditions (p < 0.0001) and diagnosed as having malaria in the last one month or the last one year (p < 0.0001) than those who did not use TM for malaria symptoms.

**Table 1:** Socio-demographic and lifestyle characteristics of adult Indonesians by the use of TM for malaria symptoms.

Characteristic	The use of TM for malaria (n = 12,226) Percentage (%) or Mean (±SD)		p-value
	Yes (n = 2,295)	No (n = 9,931)	
**Socio-demographic characteristics**			
Gender			0.0031
1. Male	54.0	50.4	
2. Female	46.0	49.6	
Area of residence			0.0422
1. Urban	24.1	20.9	
2. Rural	75.9	79.1	
Marital status			<0.0001
1. Unmarried	14.4	22.7	
2. Married	79.8	71.9	
3. Divorced	1.4	1.3	
4. Widowed	4.4	4.1	
Education			<0.0001
1. No formal	10.1	8.6	
2. Not graduated from year 6	22.1	19.9	
3. Graduated from year 6	32.6	29.3	
4. Graduated from year 9	15.5	19.6	
5. Graduated from year 12	16.3	18.6	
6. Diploma/Undergraduate	3.4	4.0	
Main occupation			<0.0001
1. Unemployed	21.9	24.9	
2. Student	3.6	6.9	
3. Officers/Police/Army	4.2	4.7	
4. Entrepreneur	13.7	14.4	
5. Farmer	43.0	33.2	
6. Fisherman	1.9	1.7	
7. Labour	5.4	6.9	
8. Other	6.2	7.1	
Per capita income			0.3508
1. Quintile 1	23.3	21.3	
2. Quintile 2	23.2	22.6	
3. Quintile 3	20.2	21.2	
4. Quintile 4	18.1	19.8	
5. Quintile 5	15.3	15.1	
**Age (year)**	40.8 (14.8)	37.6 (15.4)	<0.0001
**Lifestyle characteristics**			
Smoking/Chewing tobacco			0.0003
1. Yes, every day	38.7	33.5	
2. Yes, sometimes	6.2	6.9	
3. No, but ever smoking	5.9	5.9	
4. No, never smoking	49.2	53.7	
TM Consumption for general purposes			<0.0001
1. Yes, every day	5.9	3.4	
2. Yes, sometimes	57.0	35.8	
3. No, but ever	9.6	10.4	
4. No, never	27.5	50.5	
**BMI (kg/m**^**2**^**)**	21.7 (3.7)	21.9 (3.9)	0.0478
**Malaria status**			<0.0001
1. Diagnosed having malaria in the last one month	9.6	8.4	
2. Having malaria symptoms in the last one month	75.2	84.0	
3. Diagnosed having malaria in the last one year	2.6	0.9	
4. No malaria	12.6	6.7	

Table 2 shows the distribution of malaria prevention methods, the awareness of health service availability, and house-related variables, by the use of TM for malaria symptoms. There were no statistical associations between the use of TM for treating malaria symptoms and various methods for malaria prevention except for those who use antimalarial drugs for malaria prevention. People who used antimalarial drugs whenever they stayed overnight in a malaria endemic area were more likely to use TM for malaria symptoms (p = 0.0006) than those who did not stay overnight in malaria endemic areas. People who reported resided in areas where there were no hospitals (p = 0.0009) and no physician clinics (p = 0.0012) in their local vicinity were more likely to use TM for malaria symptoms than those participants reporting residing in local areas with hospitals and physicians clinics. Respondents who used aluminium for their home roofing (p < 0.0001), had no interior ceiling in their house (p < 0.0001) were more likely to use TM for malaria symptoms than those who used all other types of roofing and interior ceilings. Those respondents who lived near a forest area (p = 0.0024) were more likely to use TM for malaria than those who did not live near a forest area.

**Table 2:** Malaria prevention method, the awareness of health service availability, and house related variables, by the use of TM for malaria symptoms.

Malaria prevention	The use of TM for malaria (%) (n = 12,226)		p-value
	Yes (n = 2,295)	No (n = 9,931)	
**Malaria prevention**			
Sleeping under bed net			0.5110
1. Yes	52.7	51.6	
2. No	47.3	48.4	
Using coil for insecticide or using electric insecticide			0.1151
1. Yes	54.9	57.7	
2. No	45.1	42.3	
Installing mosquito screen for window			0.5400
1. Yes	11.3	11.8	
2. No	88.7	88.2	
Using mosquito repellent			0.0465
1. Yes	14.7	16.8	
2. No	85.3	83.2	
Spraying the house using insecticide			0.0132
1. Yes	14.0	16.7	
2. No	86.0	83.3	
Drinking antimalarial drug whenever staying in malaria endemic areas			0.0006
1. Yes	8.9	6.7	
2. No	91.0	93.3	
Other prevention			0.1881
1. Yes	12.2	11.0	
2. No	87.8	89.0	
**Awareness of health service availability**			
Hospital			0.0009
1. Yes	66.1	72.3	
2. No	33.9	27.7	
Community health center (*Puskesmas*/*Puskesmas* Aid)			0.8024
1. Yes	92.4	92.6	
2. No	7.6	7.4	
Physician clinic			0.0012
1. Yes	46.5	52.3	
2. No	53.5	47.7	
Midwife clinic			0.0078
1. Yes	57.3	62.0	
2. No	42.7	38.0	
Village maternal clinic (*polindes*)			0.6401
1. Yes	31.2	30.3	
2. No	68.8	69.7	
Village health post (*poskesdes*)			0.6058
1. Yes	21.6	22.4	
2. No	78.4	77.6	
Integrated village health post (*posyandu*)			0.8416
1. Yes	69.6	70.0	
2. No	30.4	30.0	
**House related variables**			
Type of the house			0.2191
1. Non-stilt house	72.4	71.4	
2. Stilt house	27.0	28.3	
3. Floating house	0.6	0.3	
Type of roofing material			<0.0001
1. Concrete	92.4	92.6	
2. Terracotta	16.2	22.0	
3. Iron wood	2.4	2.9	
4. Aluminium	60.1	58.0	
5. Asbestos	4.5	4.7	
6. Black aren fibres, dried coconut or rumbia leafs	10.2	7.8	
7. Other	3.9	2.1	
Interior ceiling material			<0.0001
1. Concrete	2.0	1.8	
2. Gypsum	1.6	2.5	
3. Asbestos	1.8	3.6	
4. Wood/Plywood	25.3	29.8	
5. Bamboo woven	3.6	4.7	
6. Other	5.5	5.8	
7. No interior ceiling	60.2	51.8	
Wall material			0.1281
1. Concrete	42.7	44.7	
2. Wood/Plywood	44.4	43.7	
3. Bamboo	10.6	8.7	
4. Aluminium	1.6	1.9	
5. Other	0.6	1.1	
Flooring material			0.3902
1. Ceramic/cement/marmer stone	33.4	35.5	
2. Broken cement	29.6	28.5	
3. Plywood/bamboo woven/bamboo/rattan	29.0	29.2	
4. Earthen floor	8.0	6.9	
Floor area (m^2^)	63.1 (66.0)	65.0 (67.7)	0.2871
House near embankment/big pond/mining areas			0.4629
1. Yes	6.2	5.6	
2. No	93.8	94.4	
House near swamp areas			0.8788
1. Yes	10.2	10.8	
2. No	89.3	89.2	
House near a river			0.9575
1. Yes	23.9	24.0	
2. No	76.1	76.0	
House near forestry areas			0.0024
1. Yes	22.2	17.7	
2. No	77.8	82.3	
House in mountainous/hilly areas			0.0059
1. Yes	26.4	22.1	
2. No	73.6	77.9	
House near beach/in coastal areas			0.0131
1. Yes	10.9	8.4	
2. No	89.1	91.6	
House in densely populated areas			0.0060
1. Yes	33.6	38.1	
2. No	66.4	61.9	
House near livestock farming areas			0.5623
1. Yes	10.7	9.9	
2. No	89.3	90.0	
House near agricultural areas			0.0770
1. Yes	33.0	30.0	
2. No	67.0	70.0	
House near wet/dry paddy field			0.0422
1. Yes	24.1	20.9	
2. No	75.9	79.1	

The output from the logistic regression model is presented in Table 3. People who identified their main occupation as farming were 1.26 (95% CI 1.08 1.47) times more likely to use TM for treating malaria, compared to those who were unemployed. People who had previously consumed TM for general health conditions were 1.84 (95% CI 1.51 2.25) times more likely to use TM for malaria symptoms, compared to people who had not previously consumed TM for general health conditions. Respondents who consumed TM for general health conditions every day (OR 3.75, 95% CI 2.93 4.79), and those who sometimes used TM for general health conditions (OR 3.23, 95% CI 2.76 3.79) were more likely to use TM for malaria symptoms, compared to those who never consumed TM for general health conditions. People who reported no hospital in their local area of residence were more likely to use TM for malaria symptoms (OR 1.31, 95% CI 1.10 1.57).

**Table 3:** Multiple logistic regression for predicting use of TM for malaria symptoms (compared with not using TM for malaria symptoms).

All variables (directions and units)	Odds ratio	95% CI	*p*-value
Main occupation			
Unemployed	1	-	-
Farmer	1.26	1.08 1.47	0.003
Student	0.85	0.64 1.13	0.262
Officer/Army/policemen	1.08	0.83 1.41	0.557
Entrepreneur	1.02	0.86 1.22	0.745
Fisherman	1.11	0.71 1.75	0.646
Labour	0.83	0.65 1.05	0.126
Other	0.94	0.75 1.19	0.639
Age (increasing years)	1.01	1.00 1.01	<0.0001
Drinking Jamu/herbs for general purposes			
No, never drink Jamu	1	-	-
Yes, every day	3.75	2.93 4.79	<0.0001
Yes, sometimes	3.23	2.76 3.79	<0.0001
No, but ever drink Jamu	1.84	1.51 2.25	<0.0001
Malaria status			
Diagnosed in 1 month	1	-	-
Having symptoms in 1 month	0.77	0.61 0.97	0.026
Diagnosed in 1 year	2.43	1.60 3.67	<0.0001
No malaria	1.46	1.10 1.93	0.009
Awareness of availability of hospital			
Yes	1	-	-
No	1.31	1.10 1.57	0.002
Roof type			
Terracotta	1	-	-
Concrete	1.61	1.14 2.26	0.006
Iron wood/shingles	1.12	0.74 1.66	0.623
Aluminium	1.61	1.35 1.92	<0.0001
Asbestos/cement	1.52	1.15 1.99	0.003
Black aren fibres/sago palm leaves (*Ijuk/rumbia*)	1.88	1.40 2.52	<0.0001
Other	2.99	2.19 4.07	<0.0001
Ceiling type			
No ceiling	1	-	-
Concrete	1.02	0.71 1.46	0.896
Gypsum	0.59	0.40 0.89	0.12
Asbestos/cement	0.46	0.30 0.71	<0.0001
Wood/Plywood	0.76	0.65 0.88	0.001
Bamboo woven	0.81	0.56 1.16	0.253
Other	0.79	0.59 1.06	0.116

Those respondents with malaria diagnosed in the last 12 months were 2.43 (95% CI 1.60 3.67) times more likely to use TM for their malaria symptoms than those who had been diagnosed as having malaria in the last month. Further, respondents who perceived themselves as having symptoms of malaria but were not diagnosed by conventional health staff were less likely to use TM for malaria symptoms (OR 0.77, 95% CI 0.61 0.97) compared to those diagnosed as having malaria in the last one month.

Study participants who lived in houses with asbestos/cement roofing (OR 1.52, 95% CI 1.15 1.99), aluminium roofing (OR 1.61, 95% CI 1.35 1.92), ijuk/rumbia (black aren fibres or sago palm leaves) roofing (OR 1.88, 95% CI 1.40 2.52), or other roofing materials (OR 2.99, 95% CI 2.19 4.07), were more likely to use TM for malaria symptoms than those who lived in houses with terracotta roofing. Likewise, people whose home had asbestos/cement ceilings (OR 0.46, 95% CI 0.30 0.71) or wood/plywood ceilings (OR 0.76, 95% CI 0.65 0.88) were less likely to use TM for malaria than those who lived in a house without a ceiling.

## Discussion

This paper reports the world-first analyses on TM use for malaria symptoms drawing upon a large sample (n = 12,226). Our research provides novel findings on TM usage by Indonesian people residing in high-risk endemic malaria areas highlighting implications for anti-malarial drug use and malaria treatment delivery in Indonesia more generally.

Approximately one in five Indonesians in our large national sample use TM for treating their malaria symptoms. This substantial prevalence rate is not dissimilar to that previously identified in nine malarial sub-districts in Purworejo, Central Java, Indonesia [14]. However, the prevalence of TM use for malaria identified in our study is far higher than the prevalence of TM use for malaria reported in rural and remote areas in Lao PDR [41], in rural-urban Thailand [42], and in rural India [43]. The relatively high prevalence rate identified in our study highlights the significance of TM use as an issue for policymakers, health services managers and conventional health practitioners in their attempts to provide safe, effective and coordinated care for those with malaria in Indonesia.

Our analyses identify those respondents from poorer households as more likely to use TM for treating malaria symptoms. This finding is in line with the results of some previous studies conducted in other low-to-middle income countries on TM use for both general health/conditions [44] and specifically for malaria treatment [45]. Having a low educational background and living in houses constructed from low-cost material have both been identified as poverty indicators [46]. In turn, both lower income and lower educational attainment are likely to lead to decreased access to conventional health care [47], and as research has shown barriers to conventional health care access may lead people to seek TM [48].

In our study, there are significant numbers of respondents with malaria symptoms who use TM despite recommended antimalarial drug treatment being provided free of charge in conventional health centers across Indonesia. Previous research has shown that in cases where pharmaceutical costs for malaria treatment are available free of charge to the patient, it is nevertheless sometimes the case that other factors (such as transportation costs associated with accessing treatment) may still impose a restrictive cost burden on some malaria patients [49]. This burden could certainly be one possible explanation for our study finding that many participants utilized TM for malaria symptoms despite free conventional anti-malarial treatments being available, especially given that TM users for malaria were more likely to be from poorer households than non-TM users for malaria. The self-treatment of malaria symptoms at home or at least within their immediate locale of residence may be a more cost-effective and, in many cases, the only viable treatment option for those with extremely limited income. Moreover, the transportation and associated costs which may be acting as a barrier to some people accessing anti-malarial drug treatment may be further accentuated by the fact that such treatment is typically initially administered over a three-day period as well as via further on-site treatment at a later date should the malaria symptoms persist. When considered alongside the fact much of Indonesia is relatively isolated with difficult terrain and/or lack of roads encountered in many areas [50–52], and that TM provision is often relatively well-represented in local communities across Indonesia, these circumstances may, in part at least, begin to help explain the substantial level of TM use amongst those with malaria symptoms despite cost-free antimalarial medications in some cases being available, albeit with variable coverage [3,53].

Another finding from our study of major significance is that TM use for malaria symptoms is higher among those respondents who have been diagnosed (by conventional health staff) as having malaria for multiple episodes in the last year when compared to those who have been diagnosed with malaria in the last month. A possible explanation for this finding may relate to the fact that patients with multiple diagnoses may experience recurrent malaria infections which are associated with a greater risk of severe and fatal malaria [54] and those experiencing recurrent infections may also exhibit poor adherence to antimalarial drug treatment. As a result, such patients may be using TM as a complement or substitution treatment for their malaria symptoms—a scenario which may further encourage low adherence which in turn can reduce the potential effectiveness of antimalarial drugs and can lead to fatal malaria as well as increase the spread of antimalarial drug resistance [55]. The high prevalence of TM use by patients with multiple episodes of malaria who have received free antimalarial drug treatment as identified in our findings highlights the significance of this under-researched health-seeking behavior for conventional malaria care and the effective delivery and promotion of antimalarial drug treatments across high-risk malaria endemic areas of Indonesia. Our data suggests there may be a pressing need to promote continuous health education around TM use and treatment options especially for those who receive or provide free antimalarial drug treatment in rural areas.

Our data shows a substantial number of those receiving a malaria diagnosis from conventional health providers are also using TM. Interestingly, previous surveys show many who receive such a medical diagnosis may also receive antimalarial drug treatment (33.7–49.1%) [7,8] and the possible concurrent use of antimalarial drug treatment with TM raises some challenges and may contribute to undermining the potential impact of providing free malaria care [52]. In other countries, an abolition of user fees without proper planning has resulted in a decrease in overall service quality and revenue as well as exacerbated difficulties in meeting recurrent expenses such as purchasing medications [56–60]. Perceptions of receiving ineffective malaria treatment (via the persistence of symptoms) have also been identified as leading some malaria patients to switch provider types across both private and public funded services as well as conventional and TM practitioners [61]. A study in Indonesia has shown some types of antimalarial drugs lead to dizziness, vomiting and nausea [62], side effects which may contribute to some patients abandoning the use of antimalarial drug treatment, and instead employing TM use which they may view as a more ‘natural’ and thereby ‘safer’ treatment option [63]. However, this issue remains under-researched, and the further empirical investigation is needed to examine the influences upon patients’ decision-making around TM use for malaria in Indonesia.

### Limitations

Our study has some limitations. The data collected was self-reported and as such may be affected by recall bias. However, the accuracy and quality of respondents’ interpretation of the questions posed were increased through the availability of an interviewer during the face-to-face data collection. The Riskesdas data set only questioned participants about their use of medicinal medicine/herbal plants, and this may have led to an underestimation of the prevalence of TM use in our analysis. Nevertheless, these limitations of the Riskesdas dataset are countered by the first opportunity to provide large-scale analyses of TM use for malaria in Indonesia. In our data set, there is no information related to an adverse effect from TM or information on the severity of malaria cases, so we were unable to consider such potential confounding factors in our analysis. The Riskesdas survey does not collect data on primaquine access and use, so we are unable to examine the use of TM among patients who received or did not receive a 14-day regimen of primaquine for preventing recurrent attacks of Plasmodium vivax. Unfortunately, detailed information on mortality and the effectiveness of TM for malaria are not available in the Riskesdas survey data, so we are unable to ascertain if death was due to malaria-related illness such as febrile illness and if the use of TM was due to the effectiveness of TM for malaria. In the next Riskesdas data, it will be useful if the Ministry of Health of Indonesia also to include measures related to user’s attitudes of TM use, the cost of treatment, quality, therapeutic success, side effects, types of products used, and main reasons for use despite the availability of free antimalarials.

## Conclusions

Our study shows that a substantial majority of people with malaria symptoms in Indonesia are utilizing TM as part of their malaria treatment alerting us to possible safety issues given the increasing but still lacking evidence-base for TM regarding malaria. Additionally, our analyses suggest many malaria patients who use TM may also be using free antimalarial drugs. Such concurrent and complementary use highlights TM as a significant issue for those looking to promote safe, effective and coordinated malaria treatment and all malaria care providers need to enquire with their patients about possible TM use and ensure adherence to antimalarial drugs where possible. There is an urgent need for further research the safety and efficacy of TM use for malaria treatment.

## Abbreviations

ACT: Artemisinin Combination Therapy
API: Annual Parasites Index
Lao PDR: Lao People’s Democratic Republic
MDGs: Millennium Development Goals
NIHRD: National Institute of Health Research Development
OR: Odd Ratio
TM: Traditional Medicine
